# Biomaterial Strategies for Three-Dimensional Bioprinting and Drug Delivery Application

**DOI:** 10.3390/ma19112186

**Published:** 2026-05-22

**Authors:** Thi Nhat Linh Phan, Thi Thuy Truong, Tan Hung Vo, Van Hiep Pham, Thi Xuan Nguyen, Thi Kim Ngan Duong, Vu Hoang Minh Doan, Jaeyeop Choi, Mrinmoy Misra, Junghwan Oh, Sudip Mondal

**Affiliations:** 1 Industry 4.0 Convergence Bionics Engineering, Department of Biomedical Engineering, Pukyong National University, Busan 48513, Republic of Korea; nhatlinh1042000@gmail.com (T.N.L.P.); thuytt2405@gmail.com (T.T.T.); ntxuanbio@gmail.com (T.X.N.); dkimngan2104@gmail.com (T.K.N.D.); 2 Smart Gym-Based Translational Research Center for Active Senior’s Healthcare, Pukyong National University, Busasn 48513, Republic of Korea; tanhung0506@gmail.com (T.H.V.); doanvuhoangminh@gmail.com (V.H.M.D.); jaeyeopchoi@pknu.ac.kr (J.C.); 3 Faculty of Mechanical Engineering and Mechatronics, PHENIKAA School of Engineering, PHENIKAA University, Nguyen Trac, Duong Noi, Hanoi 12116, Vietnam; hiep.phamvan@phenikaa-uni.edu.vn; 4 Mechatronics Engineering Department, School of Engineering, Manipal University Jaipur, Jaipur 303007, India; 5 Digital Healthcare Research Center, Institute of Information Technology and Convergence, Pukyong National University, Busan 48513, Republic of Korea

**Keywords:** 3D printing, tissue engineering, scaffolds, biomaterials, mechanical characterization, computational modelling

## Abstract

Three-dimensional (3D) bioprinting has rapidly evolved into a controlling platform for the fabrication of patient-specific biomedical implants, with growing importance in advanced drug delivery systems. Beyond structural tissue engineering, bioprinted constructs now function as programmable therapeutic depots capable of localized, sustained, and stimuli-responsive drug release. This review focuses on recent biomaterial design strategies that enable precise control over drug encapsulation, retention, and release kinetics within 3D bioprinted architectures. The physicochemical and mechanical properties of bioinks, including crosslinking density, porosity, degradation behavior, viscoelasticity, and swelling characteristics, directly influence drug loading efficiency and release dynamics under physiological conditions. The rational tuning of these parameters allows the development of constructs that provide spatially controlled and temporally regulated therapeutic delivery. Recent advances in predictive modeling, such as finite element modeling (FEM), data-driven machine learning approaches, and ML, have significantly improved the ability to correlate material composition, printing parameters, and structural geometry with drug diffusion and degradation-mediated release mechanisms. These tools facilitate the optimization of printing variables including extrusion pressure, nozzle diameter, and layer resolution to ensure structural fidelity while maintaining therapeutic functionality. Emerging strategies incorporating multi-material printing, gradient architectures, and stimuli-responsive biomaterials have expanded the potential of 3D bioprinting for combination therapies and personalized medicine. This review discusses key challenges in translating bioprinted drug delivery systems into clinical applications, including the standardization of drug release characterization methods, and long-term stability assessment.

## 1. Introduction

Three-dimensional (3D) printing has emerged as a transformative technology in biomedical engineering, offering incomparable precision in fabricating complex structures for tissue engineering, regenerative medicine, drug delivery, and personalized healthcare [1]. Among its advancements, 3D bioprinting—the layer-by-layer deposition of living cells, biomaterials, and growth factors—has attracted significant attention for its ability to replicate native tissue architecture and functionality [2]. Unlike conventional two-dimensional (2D) cell cultures, bioprinted constructs more accurately mimic the biochemical, mechanical, and microstructural properties of human tissues, enabling breakthroughs in disease modeling, drug testing, and organ regeneration [3]. The origins of 3D printing trace back to 1984 with Charles Hull’s development of stereolithography (SLA), a technique that uses photopolymerization to create layered structures [4]. Initially employed for industrial prototyping, the technology quickly found biomedical applications. The transition to bioprinting began in the mid-1990s, driven by advances in biomaterials and cell biology, including Dr. Gabor Forgacs’ pioneering work on cell self-assembly into 3D structures [5]. By the early 2000s, researchers had developed the first synthetic organ scaffolds colonized with patient-derived cells, marking a critical milestone in regenerative medicine [6]. Innovations such as hybrid bioprinters, which combine multiple deposition methods, and smart bioinks, which respond to environmental stimuli, have further expanded the field’s capabilities. More recently, advances in multi-material bioprinting, 4D bioprinting (where printed structures evolve over time), and the AI-driven optimization of printing parameters have pushed the boundaries of what is possible in tissue engineering. The bioprinting process typically involves three stages: pre-processing, where a digital model is generated from medical imaging or computer-aided design (CAD); processing, where bioinks are deposited layer-by-layer; and post-processing, where constructs are matured in bioreactors to promote tissue development [7]. Several bioprinting technologies are employed, each with distinct advantages. Extrusion-based bioprinting is versatile and suitable for large-scale tissues but has limited resolution [8]. Inkjet bioprinting offers the high-speed deposition of low-viscosity bioinks, while laser-assisted bioprinting (LAB) provides high resolution, making it ideal for delicate cell types [9]. SLA uses light-based photopolymerization to create intricate structures with excellent precision [10]. The choice of technique depends on the specific application, balancing factors such as resolution, scalability, and biocompatibility.

Biomaterials serve as the foundation for bioprinted tissues and can be broadly categorized as natural, synthetic, or composite. Natural biomaterials, such as collagen, gelatin, and hyaluronic acid, are derived from biological sources and offer high biocompatibility, closely mimicking the extracellular matrix (ECM) [11]. Their mechanical properties are often limited. Synthetic biomaterials, including polylactic acid (PLA) and polycaprolactone (PCL), provide superior mechanical strength and tunable degradation rates but lack innate bioactivity [12]. Composite biomaterials combine natural and synthetic components to achieve a balance of biocompatibility, mechanical integrity, and functionality [13]. Key properties of biomaterials include biocompatibility, mechanical strength, biodegradability, and bioactivity, all of which must be carefully optimized to ensure successful tissue integration and function. The present study methodology was partially aligned with the systematic literature review (SLR) approach reported by Maedeh Amirpour et al., where online databases such as Scopus and Google Scholar were systematically used to identify and optimize relevant publications through specific keyword combinations. A large number of manuscripts related to biomaterials, 3D printing, and scaffold engineering were initially identified; however, the most relevant studies were selected based on three major criteria: (1) biomaterials with 3D printing properties, (2) scaffold-based drug delivery applications, and (3) the mechanical stability of 3D-printed scaffolds. Unlike conventional bibliographic studies that mainly focus on publication trends and network analysis, our review emphasized clinically applicable biomaterial strategies for tissue regeneration and regenerative medicine. This approach allowed us to critically analyze how scaffold design, material selection, and drug delivery integration can contribute to the development of mechanically stable and functional scaffolds for future biomedical and clinical applications [13].

**Figure 1:**
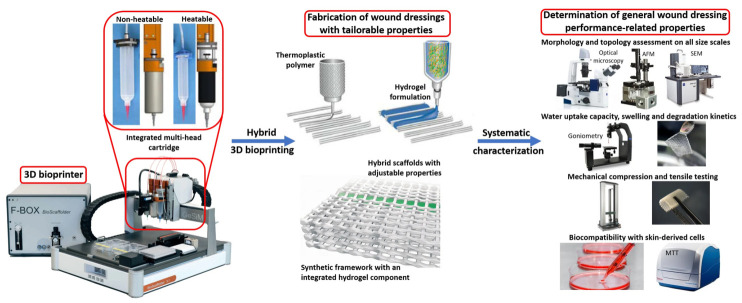
Schematic of hybrid 3D bioprinting for fabrication of wound dressings with tunable properties and their subsequent physicochemical, mechanical, and biocompatibility characterization, reproduced with permission from [14].

Figure 1 illustrates the hybrid extrusion-based 3D bioprinting strategy developed to overcome the limited mechanical stability of conventional polysaccharide hydrogels used in regenerative medicine. By enabling the simultaneous printing of rigid thermoplastic polymers and soft hydrogels at different temperatures, the layer-by-layer fabrication process produces hybrid scaffolds with integrated hydrogel components and enhanced mechanical performance. The resulting structures exhibit tunable physicochemical and biological properties, including controllable wettability, swelling behavior, biodegradability, mechanical strength, diverse surface morphologies, and favorable biocompatibility, making them promising for tissue engineering and wound dressing applications [14].

Beyond tissue engineering, 3D bioprinting has significantly advanced drug testing and disease modeling. Bioprinted organ-on-a-chip platforms provide physiologically relevant environments for studying disease mechanisms and evaluating drug responses [15]. Personalized medicine has also benefited, with patient-specific implants and prosthetics tailored to individual anatomical needs [16]. In addition, bioprinted tumor models are increasingly used to investigate cancer progression and assess individualized therapeutic strategies. A comparative overview of commonly used biomaterials, including their key physicochemical properties and typical biomedical applications, is summarized in Table 1, highlighting the relationship between material characteristics and their functional performance in diverse biomedical contexts.

Looking ahead, the field aims to overcome challenges such as vascularization, scalability, and regulatory approval to enable the fabrication of fully functional complex organs. Continued progress is driven by innovations in smart biomaterials, real-time monitoring systems, and artificial intelligence (AI)-assisted optimization. Stimuli-responsive biomaterials may enable dynamic tissue remodeling, while integrated sensing systems could facilitate the real-time monitoring of tissue development [17]. Despite these advances, significant challenges remain, including the need for improved vascularization strategies, standardized regulatory frameworks, and cost-effective manufacturing approaches. Addressing these challenges will require strong interdisciplinary collaboration to fully realize the potential of bioprinting in regenerative medicine and personalized healthcare.

**Table 1:** Materials in 3D Printing for Biomedical Applications.

Material Type	Example	Properties	Common Applications	Ref.
Polymers				
Thermoplastics	PLA, ABS, PETG, TPU, Nylon	Lightweight, durable, flexible, easy to print	Prototyping, mechanical parts, wearables	[18]
Photopolymers	Resins (Standard, Tough, Flexible)	High resolution, smooth surface finish, biocompatible options	Dental modals, biomedical devices, tissue engineering scaffolds	[19]
Elastomers	TPE, TPU, Silicone	High elasticity, flexibility, and tear resistance	Soft robotics, prosthetics, seals, gaskets	[20]
Metals				
Stainless Steel	316L, 17-4 PH	High strength, corrosion resistance, biocompatible	Medical implants (e.g., bone crews, stents), tooling	[21]
Titanium	Ti-6Al-4V	High strength-to-weight ratio, biocompatible, corrosion-resistant	Aerospace, medical implants (e.g., bone plates, dental implants)	[22]
Aluminum	AlSi_10_Mg	Lightweight, good thermal and electrical conductivity	Automotive parts, lightweight biomedical devices	[23]
Cobalt-Chrome	CoCr	High wear resistance, biocompatible, high-temperature stability	Dental implants, orthopedic implants, turbine blades	[24]
Ceramics				
Alumina	Al_2_O_3_	High hardness, thermal stability, biocompatible	Dental crowns, bone scaffolds, cutting tools	[25]
Zirconia	ZnO_2_	High strength, facture toughness, biocompatible	Dental implants, orthopedic implants, wear resistant components	[26]
Hydroxyapatite	Ca_10_(PO_4_)_6_(OH)_2_	Biocompatible osteoconductive (promotes bone growth)	Bone tissue engineering dental implants	[27]
Composite				
Carbon Fiber Reinforced	Carbon fiber + Nylon, PLA, PEEK	High strength-to-weight ratio, stiffness, durability	Aerospace, automotive, sports equipment	[28]
Glass Fiber Reinforced	Glass fiber + Nylon, ABS	High strength, impact resistance, lightweight	Automotive parts, enclosures, structural components	[29]
Ceramic-Polymer	Ceramic particles + Photopolymer	Biocompatible, high resolution, tailored mechanical properties	Dental models, biomedical devices, tissue engineering scaffolds	[30]
Biomaterials				
Hydrogels	Alginate, Gelatin, Hyaluronic Acid	Biocompatible, tunable mechanical properties, cell-friendly	Bioprinting, tissue engineering, drug delivery systems	[31]
Decellularized ECM	dECM from heart, liver, cartilage	Biocompatible, biomimetic, promotes cell growth and tissue formation	Tissue engineering, regenerative medicine	[32]
Bioinks	Cell-laden hydrogels, PEG-based	Biocompatible, printable, supports cell viability and function	Bioprinting, organ-on-a-chip models, regenerative medicine	[33]
Advanced functional biomaterials				
Conductive Polymers	PEDOT:PSS, Conductive PLA	Electrically conductive, flexible, printable	Wearable electronics, sensors, flexible circuits	[34]
Magnetic Materials	Magnetic PLA, Iron-filled Nylon	Magnetic properties, good mechanical strength	Sensors, actuators, robotics	[35]

## 2. Overview of 3D Printing Technologies

Three-dimensional printing, also known as additive manufacturing, has revolutionized the fabrication of complex structures with precise control over geometry, material composition, and functionality. In the context of biomedical applications, 3D printing technologies enable the creation of patient-specific implants, tissue scaffolds, and drug delivery systems. This section provides an overview of the most widely used 3D printing technologies, with a focus on their relevance to biomaterials and bioprinting.

### 2.1. Types of 3D Printing Technologies

#### 2.1.1. Fused Deposition Modeling (FDM)

Fused Deposition Modeling (FDM), also known as Fused Filament Fabrication (FFF), is one of the most widely used additive manufacturing (AM) techniques due to its simplicity, cost-effectiveness, and versatility [36]. The process involves extruding thermoplastic materials through a heated nozzle, which deposits the material layer by layer onto a build platform to create a three-dimensional object [36]. The FDM process begins with a digital 3D model, typically designed using CAD software. This model is then sliced into thin horizontal layers using specialized software, generating a G-code file that directs the printer’s movements [37]. During printing, a thermoplastic filament is fed into an extruder, heated to a semi-molten state, and deposited onto the build platform according to the G-code instructions [38]. As the material cools, it solidifies and bonds with previously deposited layers, forming a cohesive structure. This layer-by-layer approach enables the fabrication of complex geometries with high precision, making FDM ideal for customized biomedical devices.

Material selection is critical in FDM, as it directly influences the mechanical, thermal, and biomedical properties of the final product. The most commonly used thermoplastics include PLA, a biodegradable and biocompatible polymer derived from renewable resources. PLA is favored for its low melting temperature (180–220 °C), ease of processing, and ability to support cell adhesion, making it suitable for tissue engineering and low-cost medical applications [39]. Acrylonitrile Butadiene Styrene (ABS) offers superior strength and durability, making it ideal for prosthetics and medical devices, though it requires a heated build platform to prevent warping [40]. PCL is another key material, particularly for tissue engineering scaffolds and drug delivery systems, due to its low melting point (60 °C), flexibility, and slow degradation rate [41]. Polyethylene Terephthalate Glycol (PETG) is also widely used for its transparency, chemical resistance, and ease of sterilization, making it suitable for medical devices and packaging [42]. Composite materials, such as carbon fiber-reinforced PLA, enhance mechanical properties for specialized biomedical applications. The mechanical and structural properties of FDM-printed parts are influenced by several process parameters that must be carefully optimized [43]. Nozzle temperature affects material viscosity and flowability, impacting layer adhesion and surface finish. Build platform temperature is crucial to prevent warping and ensure the proper adhesion of the first layer. Layer thickness determines resolution and surface quality, with thinner layers yielding higher precision but longer print times. Printing speed must be balanced to avoid defects: high speeds may compromise layer adhesion, while low speeds improve accuracy but extend production time. Infill density and pattern (e.g., rectilinear, honeycomb) also play a significant role in determining the strength and weight of the printed part [44]. Customized prosthetics and orthotics can be produced at a fraction of traditional costs, while surgical guides and anatomical models enhance preoperative planning. FDM is also employed in drug delivery systems, where biodegradable polymers like PCL and PLA enable controlled drug release.

#### 2.1.2. Stereolithography (SLA)

Stereolithography (SLA), one of the earliest and most precise additive manufacturing (AM) technologies, was introduced by Charles Hull in the 1980s. As a vat photopolymerization technique, SLA uses ultraviolet (UV) light to selectively cure liquid photopolymer resins layer by layer, producing highly detailed 3D structures [4]. Initially used for rapid prototyping, SLA has evolved into a versatile manufacturing tool with applications in aerospace, automotive, and consumer goods. In recent years, it has gained significant traction in biomedical engineering due to its unmatched resolution and surface finish, making it ideal for bioprinting, tissue engineering scaffolds, drug delivery systems, and patient-specific medical implants. The SLA process begins with a digital 3D model, which is sliced into thin cross-sectional layers using specialized software [45]. A UV laser or digital light projector (DLP) then selectively cures the liquid resin, solidifying each layer through photopolymerization. After each layer is cured, the build platform adjusts vertically, allowing the next layer to be formed. This iterative process continues until the complete object is fabricated. Key components of an SLA system include the UV light source, photopolymer resin (composed of monomers, oligomers, and photo-initiators), the build platform, and a control system that translates the 3D model into machine instructions [46]. The technology’s ability to achieve resolutions as fine as 10–50 µm enables the fabrication of intricate microstructures essential for mimicking biological tissues. SLA offers high resolution, smooth finishes, and compatibility with various biocompatible resins, making it valuable for implants, tissue scaffolds, surgical guides, and microfluidic devices [10]. Advances include bioactive resins, multi-material printing, and high-speed systems like DLP and CLIP [47]. Challenges remain, such as limited biocompatible materials, cytotoxicity risks, post-processing complexity, and restrictions on heterogeneous structures. Ongoing innovations in materials and hybrid printing aim to overcome these limitations for broader biomedical applications [48].

#### 2.1.3. Selective Laser Sintering (SLS)

Selective Laser Sintering (SLS) is a powder-based additive manufacturing technology that uses a high-powered laser to fuse powdered materials into solid 3D structures. Developed in the 1980s at the University of Texas at Austin, SLS has become a valuable manufacturing method due to its ability to create complex geometries without support structures [49]. Unlike other AM technologies like FDM or SLA, SLS uses unsintered powder as natural support, enabling intricate designs that are difficult to achieve with traditional methods [50]. In biomedical engineering, SLS has gained attention for processing various materials—polymers, ceramics, and composites—making it suitable for fabricating customized biomaterials, tissue scaffolds, and medical devices. The SLS process begins with a digital 3D model sliced into thin layers. A laser selectively sinters powdered material layer by layer, while the build platform lowers to deposit fresh powder for each new layer [51]. Unsintered powder acts as support, eliminating the need for additional structures. Key components include the laser source, powder bed, powder delivery system, and control system. With layer thicknesses typically between 50–100 µm, SLS achieves fine details and complex geometries, making it ideal for biomedical applications requiring precision and material versatility [52]. SLS enables the fabrication of complex, support-free structures with high mechanical strength, ideal for tissue scaffolds, load-bearing implants, and patient-specific devices. Its wide material compatibility allows the tailoring of mechanical and biological properties, while unused powder can be recycled for efficiency. Key challenges include limited biocompatible powders, high sintering temperatures, surface roughness, and high costs. Advances such as bioactive powders, multi-material systems, faster lasers, and hybrid bioprinting are expanding SLS’s potential in tissue engineering, drug delivery, and regenerative medicine [53].

#### 2.1.4. Bioprinting-Specific Methods

Bioprinting represents a revolutionary frontier in regenerative medicine, enabling the precise fabrication of living tissues through the layer-by-layer deposition of bioinks—specialized formulations containing living cells, biomaterials, and bioactive molecules [54]. This advanced manufacturing approach aims to replicate the intricate architecture and functionality of native human tissues, offering transformative potential for addressing critical healthcare challenges including organ shortages, personalized medicine, and more predictive drug testing platforms. While the technology has demonstrated remarkable progress in creating various tissue constructs, significant challenges remain in achieving vascularization, ensuring long-term cell viability, and scaling production for clinical applications. The field encompasses several distinct bioprinting modalities, each with unique capabilities and limitations. Extrusion-based bioprinting, currently the most widely adopted approach, utilizes pneumatic or mechanical dispensing systems to deposit high-viscosity bioinks, making it particularly suitable for creating dense tissue constructs such as cartilage and skin grafts [55]. The mechanical stresses inherent in the extrusion process can compromise cell viability, driving ongoing innovations in nozzle design and bioink formulations to mitigate these effects. Alternative approaches like inkjet bioprinting offer gentler cell deposition through droplet-based mechanisms, enabling high-resolution patterning ideal for creating intricate tissue architectures, though limited by the need for low-viscosity bioinks that may lack structural integrity [56]. Advanced techniques such as laser-assisted bioprinting achieve single-cell resolution via laser-induced forward transfer, enabling the creation of complex microtissues and vascular networks. Light-based stereolithography bioprinting cures cell-laden hydrogels with micron-scale accuracy, though limited by available biocompatible photoactive materials [57]. These methods complement each other, with choice determined by the specific tissue application. A key challenge in clinical translation is developing bioinks that balance printability, structural integrity, and bioactivity. Researchers are designing advanced hydrogels incorporating polymers, decellularized matrix, and nanomaterials to better replicate native microenvironments [58]. Vascularization remains critical, and strategies such as sacrificial printing, microfluidics, and self-assembling endothelial cells show promise. Applications range from patient-specific grafts and regenerative scaffolds to physiologically relevant drug-testing platforms [59]. While fully functional organ printing is still distant, ongoing advances in complexity, vascularization, and biomaterial design are steadily moving the field forward.

### 2.2. Current Trends in Bioprinting

#### 2.2.1. Innovations in Bioprinting Technologies

Recent advances in hardware, software, and process optimization are enabling the bioprinting of increasingly complex and biologically relevant tissues. High-resolution technologies, such as digital light processing (DLP) and two-photon polymerization (2PP), can replicate the intricate microarchitecture of native tissues, including vascular networks and porous bone structures [60]. DLP uses projected UV light to cure bioinks layer by layer with micrometer precision, while 2PP employs femtosecond lasers to achieve sub-micron resolution for microtissues and organ-on-a-chip devices [61]. These innovations are expanding bioprinting’s potential in tissue engineering and regenerative medicine. Multi-material bioprinting enables the fabrication of heterogeneous tissue constructs by depositing different materials with varied mechanical and biological properties. Techniques such as multi-nozzle extrusion and multi-channel inkjet printing have been used to create osteochondral scaffolds and vascularized tissues, mimicking native structural transitions [62]. Hybrid bioprinting combines complementary methods—such as extrusion with inkjet or laser-assisted with stereolithography—to merge mechanical strength, precision, and cell viability [63]. These approaches are advancing the creation of complex, functional tissues and bringing the field closer to fabricating multi-tissue organs. In addition to hardware innovations, software advancements are playing a crucial role in the evolution of bioprinting technologies. Advanced CAD and simulation tools are enabling researchers to design and optimize bioprinted structures with unprecedented precision. For example, computational fluid dynamics (CFD) simulations can be used to model the flow of bioinks during the printing process, optimizing nozzle design and printing parameters to minimize shear stress on cells [64]. Similarly, FEM can be used to predict the mechanical behavior of bioprinted structures, ensuring that they meet the requirements of specific applications. These software tools are not only improving the quality and reproducibility of bioprinted constructs but also accelerating the design and development process [65].

The integration of artificial intelligence (AI) and machine learning (ML) into bioprinting workflows is an emerging trend with significant potential. AI and ML algorithms can analyze large datasets to identify optimal bioink formulations, printing parameters, and post-printing culture conditions. For example, machine learning models can predict cell viability and tissue formation based on input parameters such as bioink composition, printing speed, and laser intensity. These predictive models can guide the optimization of bioprinting processes, reducing trial and error and improving outcomes. As AI and ML technologies continue to evolve, they are expected to play an increasingly important role in advancing bioprinting research and applications.

#### 2.2.2. Integration of Biomaterials and Living Cells

The integration of biomaterials and living cells is a cornerstone of bioprinting, enabling the fabrication of biologically functional tissues and organs. Biomaterials serve as the structural and biochemical foundation of bioprinted constructs, providing mechanical support, biochemical cues, and a conducive environment for cell growth and differentiation. Meanwhile, living cells are the building blocks of tissues, responsible for tissue formation, remodeling, and function. The successful integration of biomaterials and living cells requires the careful consideration of material properties, cell compatibility, and printing parameters. Below, we explore current trends in the development of biomaterials, bioinks, and cell-based strategies for bioprinting. One of the most significant trends in biomaterials for bioprinting is the development of bioactive and biomimetic materials. Native tissues are composed of a complex ECM that provides structural support, biochemical cues, and mechanical signals to cells. To replicate this environment, researchers are developing biomaterials that mimic the composition, structure, and function of the native ECM. For example, decellularized ECM (dECM) derived from tissues such as heart, liver, and cartilage is being used as a bioink component [66]. The dECM retains the natural biochemical and structural properties of the tissue, providing an ideal environment for cell growth and tissue formation. Similarly, synthetic biomaterials, such as PEG and PCL, are being functionalized with bioactive molecules, such as peptides, growth factors, and cytokines, to enhance their bioactivity [67]. These biomimetic materials are enabling the fabrication of more biologically relevant tissues, improving outcomes in tissue engineering and regenerative medicine. Dynamic and responsive biomaterials mimic the adaptive nature of native tissues by altering their properties in response to stimuli such as temperature, pH, or mechanical forces [68]. Examples include stimuli-responsive hydrogels and strain-stiffening materials that provide cells with appropriate biochemical and mechanical cues. Advanced bioinks are also transforming bioprinting by improving printability, cell viability, and bioactivity. Composite bioinks combining synthetic strength with natural bioactivity, along with cell-laden formulations containing multiple cell types, enable the creation of complex, functional tissues. These innovations are expanding the potential for fabricating biologically relevant and clinically applicable tissue constructs. Cell-based strategies, including the use of induced pluripotent stem cells (iPSCs), cell spheroids, and organoids, are advancing the creation of complex and functional bioprinted tissues. iPSCs can differentiate into diverse cell types, while spheroids and organoids replicate native tissue architecture and function [69]. Post-printing culture and maturation in bioreactor systems further enhance tissue development by providing mechanical, chemical, and biological cues [70]. Perfusion bioreactors support vascular network maturation, and mechanical stimulation promotes the functionality of muscle and cartilage constructs. Together, these approaches are bringing bioprinting closer to producing fully functional, clinically relevant organs.

## 3. Biomaterials in 3D Printing

### 3.1. Categories of Biomaterials

The field of biomaterials encompasses a wide and ever-expanding range of materials. These include everything from soft, cell-supporting hydrogels, mechanically robust metals and ceramics, as well as nanoscale systems such as nanoparticles and quantum dots for drug delivery and bioimaging. It also extends to advanced implantable medical devices, such as artificial hearts and left ventricular assist devices. With ongoing advances in materials science, chemistry, and biotechnology, the design and functionality of biomaterials continue to expand, enabling increasingly sophisticated biomedical applications.

**Figure 2:**
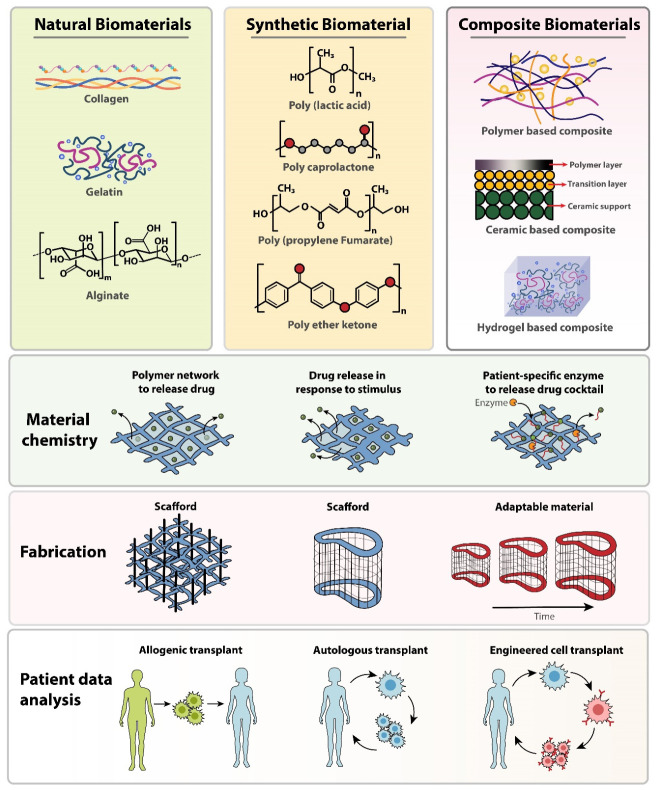
An overview of biomaterials categorizes them into three main types and their application.

Biomaterials can be broadly classified into three primary categories: natural, synthetic, and composite biomaterials, each offering distinct advantages and limitations, as illustrated in Figure 2. Natural biomaterials, such as collagen and gelatin, are derived from biological sources and exhibit excellent biocompatibility and bioactivity, closely mimicking the native extracellular matrix (ECM) [71]. These properties make them highly suitable for promoting cell adhesion, proliferation, and tissue regeneration. However, their relatively poor mechanical strength and rapid degradation often require chemical modification or crosslinking to enhance stability for long-term applications [72].

Synthetic biomaterials, including PLA and PCL, provide superior mechanical properties, tunable degradation rates, and reproducible fabrication characteristics. Their chemical structures, as shown in Figure 2, can be precisely engineered to tailor properties such as hydrophobicity, crystallinity, and mechanical strength. However, their lack of intrinsic bioactivity and the need for processing conditions such as elevated temperatures or organic solvents may limit direct cell encapsulation, often requiring post-fabrication cell seeding or surface functionalization strategies [73].

Composite biomaterials combine natural and synthetic components to bridge the gap between bioactivity and mechanical performance. By integrating organic polymers with inorganic phases, such as bioactive ceramics or nanoparticles, these materials can achieve enhanced structural integrity while maintaining a biologically favorable microenvironment. Such hybrid systems are particularly promising for load-bearing tissue engineering and multifunctional biomedical applications [74].

Beyond material classification, material chemistry plays a critical role in determining the physicochemical and biological performance of biomaterials. Parameters such as molecular structure, functional groups, crosslinking density, and degradation mechanisms directly influence biocompatibility, mechanical behavior, and cellular responses. These chemical characteristics must be carefully tailored to match the requirements of specific tissues and applications [75].

Equally important are fabrication strategies, which dictate the final architecture and functionality of bioprinted constructs. Techniques such as extrusion-based, inkjet, and laser-assisted 3D bioprinting enable precise control over scaffold geometry, porosity, and the spatial distribution of cells and bioactive agents. The interplay between material properties and fabrication parameters is critical for achieving high-resolution structures with adequate mechanical stability and biological performance [76].

In addition, the integration of patient-specific data is emerging as a key factor in advancing personalized medicine. Medical imaging modalities, such as computed tomography (CT) and magnetic resonance imaging (MRI), can be used to generate patient-specific anatomical models, enabling the design of customized implants and tissue constructs [77]. Furthermore, biological data, including genetic and cellular information, can inform the selection and optimization of biomaterials to improve therapeutic outcomes and reduce the risk of immune rejection.

Despite significant advancements in 3D printing technologies, several challenges remain before widespread clinical adoption can be achieved. These include issues related to standardization, the integration of bio-fabrication platforms, software development, printer capabilities, reproducibility, quality control, biomaterial characterization, and regulatory approval. Biomaterial inks must be scalable, cost-effective, and readily available for clinical translation. Their suitability depends on both the selected printing technique and the intended application, requiring a careful balance of mechanical, biological, and processing properties to achieve optimal performance [78].

Three-dimensional bioprinting holds great promise for transforming healthcare by enabling personalized treatment strategies, improving patient outcomes, and supporting on-demand fabrication in clinical settings. By tailoring biomaterials to specific medical needs, this technology can enhance therapeutic efficacy and patient compliance. This review aims to provide a comprehensive analysis of currently available 3D-printable biomaterials, highlighting their advantages, limitations, and future potential in biomedical applications. Ultimately, it seeks to guide ongoing research and inspire the development of next-generation biomaterials for advanced healthcare solutions. A summary of the key properties of the biomaterials is presented in Table 2.

#### 3.1.1. Natural Biomaterials

*a. Collagen*

Collagen, abundant in the ECM of various tissues, is a popular natural material used in cell and tissue culture. As the main component of musculoskeletal tissue, this biocompatible, triple-helical protein, derived naturally, minimizes immune responses in scaffolds and promotes cell growth, adhesion, and attachment [31]. Established methods for extracting and purifying type I collagen have led to its widespread use in creating cell culture surface coatings and gels. The arginine-glycine-aspartic acid (RGD) sequences within collagen fibers promote cell proliferation and attachment. Despite its use in bioprinting, type I collagen has limitations. It remains liquid at low temperatures, forming fibrous structures only upon warming, with complete gelation taking up to 30 min at 37 °C, hindering the printing of stable 3D structures. A 2017 study by Nicole Diamantides et al. explored the impact of riboflavin photo-crosslinking and pH on the rheological properties and printability of collagen [79]. Their findings indicated that while the gelation rate did not influence printability, the shape fidelity of printed constructs during collagen gelation was highly pH dependent.

While collagen is crucial to native tissue and ECM, combining it with other ECM components is being explored to improve its function. Pure collagen matrices, though biologically advanced, may not always be ideal. Lacking components like elastin, glycosaminoglycans (GAGs), fibrinogen, and laminin could disrupt cellular signaling. The hydrophobicity of collagen gels can limit water absorption, nutrient and gas exchange, and cell viability in implants or cell carriers. Therefore, creating hybrid biomaterials that incorporate collagen with other ECM components could lead to better tissue engineering outcomes.

*b. Gelatin*

Gelatin, a collagen-derived protein, exhibits amphoteric behavior due to its alkaline and acidic amino acid functional groups [83]. It is commonly sourced from animal connective tissues and has been widely used in regenerative medicine due to its biocompatibility, biodegradability, water solubility, and low immunogenicity. These properties make gelatin methacryloyl (GelMA) a preferred material for direct ink writing (DIW) bioprinting. Recent studies, such as those by Lee et al. [80], have compared different GelMA formulations for cell-laden bioprinting, reporting up to 75% cell viability, while Liu et al. [84] demonstrated that highly porous, low-stiffness GelMA structures effectively support cell survival and proliferation. Unlike native collagen, gelatin dissolves in neutral pH solutions while maintaining its ability to form linear gels through hydrophobic cross-linking at low temperatures. Since gelatin gels melt between 30 °C and 35 °C, additional modifications like chemical crosslinking or polymer blending are often required for biomedical applications. For example, gelatin-fibrinogen crosslinked with glutaraldehyde has been used with dermal fibroblasts to develop wound-healing scaffolds that promote collagen production, cell infiltration, and biodegradation. Electrospun gelatin–PCL nanofibers have also been studied for skin tissue engineering, demonstrating enhanced wound healing and re-epithelialization. Gelatin-based platforms have been engineered to deliver growth factors, such as basic fibroblast growth factor (bFGF), facilitating rapid tissue regeneration and vascularization. Due to its thermal sensitivity and functional versatility, gelatin has been widely explored in bioprinting, with modifications enabling controlled degradation, improved mechanical stability, and enhanced cellular interactions.

*c. Alginate*

**Figure 3:**
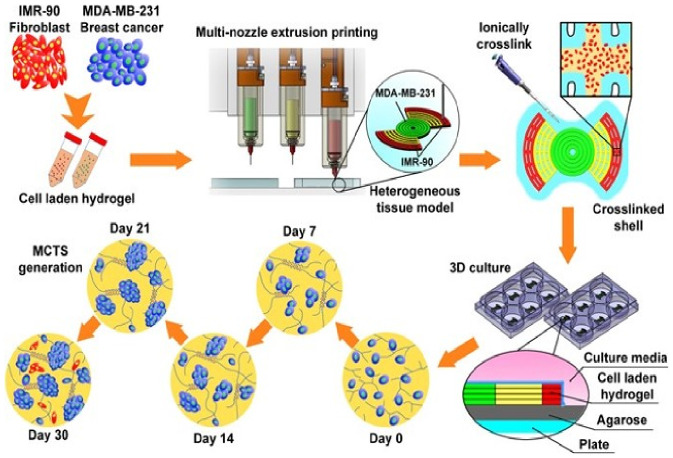
A schematic illustrating the design and experimental approach for developing a heterogeneous tumor model containing MDA-MB-231 triple-negative breast cancer cells and IMR-90 fibroblasts, reproduced with permission from [81].

Alginate is a naturally occurring, water-soluble polysaccharide derived from the cell walls of various brown algae species, such as Laminaria, Macrocystis, and Ascophyllum. As a biomaterial, alginic acid and its sodium and potassium salts (ALG) are widely used due to their biocompatibility, biodegradability, chemical versatility, and ability to facilitate sol–gel transitions. Among natural hydrogels, alginate is one of the most commonly used bioinks in 3D bioprinting because of its affordability, ease of crosslinking, and compatibility with various printing techniques, including inkjet and direct ink writing (DIW). Its low cell adhesion compared to other natural polymers presents a challenge. To address this, researchers have combined alginate with other biopolymers like gelatin and fibrinogen to enhance cell interaction. Several studies have demonstrated alginate’s potential in tissue engineering, drug delivery, and cell encapsulation. Hong et al. [85] present a 3D-printable, highly stretchable hydrogel made from PEG and sodium alginate, exhibiting superior toughness compared to natural cartilage. The hydrogel supports high cell viability for over seven days and enables the fabrication of intricate 3D structures without extra support materials, thanks to the addition of biocompatible nanoclay. Its exceptional mechanical properties stem from the synergy between reversible alginate crosslinking and PEG elasticity, providing both durability and flexibility. This study marks a notable advancement in hydrogel technology, offering a robust and biocompatible material for biomedical and tissue engineering applications. Jiang et al. [81] propose a 3D bioprinting approach for the fabrication of biomimetic tumor models by encapsulating MDA-MB-231 breast cancer cells and IMR-90 fibroblasts within an alginate–gelatin hydrogel matrix, as shown in Figure 3. Over a 15-day period, fibroblasts migrate and integrate into tumor spheroids, resulting in the formation of heterogeneous cell structures that remain viable for extended durations. The composite hydrogel exhibits tunable mechanical properties, facilitating prolonged cell culture (>30 days) and controlled cellular interactions. This bioprinted model provides a robust platform for studying tumor progression, cell–cell interactions, and therapeutic interventions within a physiologically relevant microenvironment.

*d. Fibrin*

Fibrin, a fibrous protein derived from fibrinogen, plays a crucial role in various biomedical applications due to its excellent biocompatibility, biodegradability, and mechanical properties. Primarily synthesized in the liver, fibrinogen is found in blood plasma, platelets, lymph, and interstitial fluid [86]. Upon vascular injury, thrombin hydrolyzes fibrinogen, leading to fibrin polymerization, which is essential for blood clot formation. Beyond its natural function, fibrin serves as a valuable biomaterial in drug delivery, tissue engineering, and regenerative medicine [87]. Its rapid gelation, ability to promote cell growth and angiogenesis, and strong binding affinity to extracellular matrix proteins make it an ideal scaffold material for engineering tissues such as skin, blood vessels, and bone. Fibrin is widely used as a surgical sealant and has gained prominence in bioprinting for creating scaffold-free tissue structures. Despite its advantages, fibrin remains an expensive material, necessitating further research into cost-effective production methods [88]. Its adaptability and functional properties continue to drive innovation in biomedical and tissue engineering applications. Hakam et al. [88] developed a fibrin–gelatin hybrid hydrogel as a biopaper for skin bioprinting. The optimized hydrogel showed excellent absorption, biodegradability, mechanical strength, and supported fibroblast-based bioink, promoting tissue fusion. This highlights its potential for skin bioprinting applications. An another study by England et al. [89] introduced a novel bioprinting method to fabricate fibrin-based scaffolds, overcoming extrusion-based 3D printing challenges caused by fibrinogen’s low viscosity. By using hyaluronic acid (HA) and polyvinyl alcohol (PVA) for viscosity control and factor XIII for crosslinking, they created 3D fibrin-factor XIII-HA scaffolds without support structures. These scaffolds effectively encapsulated Schwann cells, promoting their viability, proliferation, and alignment for nerve regeneration. The induced fiber and cellular alignment also make this technique suitable for regenerating aligned tissues like muscle, skin, and vasculature, providing a biomimetic alternative to synthetic hydrogels.

#### 3.1.2. Synthetic Biomaterials

*a. Poly(lactic acid)*

PLA is one of the most widely used polymers in 3D printing for biomedical applications due to its biocompatibility, biodegradability, cost-effectiveness, and ease of processing [90]. Derived from renewable sources such as corn and wheat, PLA offers a sustainable alternative to petroleum-based polymers like ABS and nylon. Its thermoplastic nature allows for easy fabrication using various additive manufacturing techniques, including FDM, material extrusion (MEX), and binder jetting (BJT). In tissue engineering and regenerative medicine, PLA is extensively utilized in the creation of customized scaffolds, particularly for bone tissue regeneration. Its inherent brittleness and the release of acidic byproducts during degradation pose challenges for long-term biocompatibility. To address this, PLA–ceramic composites, especially with calcium phosphates, have been developed to enhance mechanical strength, mineralization, and biocompatibility. Beyond scaffolds, 3D-printed PLA plays a crucial role in drug delivery systems, enabling the controlled release of therapeutic agents such as antibiotics, chemotherapeutics, and hormones. PLA is widely used in the fabrication of diagnostic electrodes, prosthetic devices, orthoses, surgical instruments, radiotherapy devices, and training models. Its ability to produce intricate, patient-specific medical constructs has significantly advanced personalized medicine and healthcare innovation. Tcacencu et al. [82] developed a 3D-printed scaffold combining PLA and apatite–wollastonite (AW/PLA) using material extrusion (MEX) and binder jetting (BJT) to enhance bone regeneration. This composite effectively mimics cortical and cancellous bone structures, demonstrating biocompatibility, osseointegration, and osteoinductive properties. In vitro, all scaffold types supported cell proliferation and osteogenic differentiation, while in vivo, the AW/PLA scaffold exhibited greater bone formation (~20%) and vascularization in a rat calvarial defect model, significantly surpassing plain PLA (~2%) after 12 weeks. With strong initial bonding, slow degradation, and improved bone ingrowth, this hybrid scaffold minimizes delamination, highlighting macro-scale composite scaffolds as a promising solution for bioactive bone implants. Liu et al. introduce an innovative strategy for addressing large bone defects by utilizing a 3D-printed polylactic acid–hydroxyapatite (PLA-HA) scaffold infused with enhanced bone marrow (eBM) and integrated with induced membrane (IM) [91]. In vitro studies confirmed the biocompatibility of the PLA-HA scaffold and the osteogenic potential of eBM-derived mesenchymal stem cells (MSCs). In vivo, a rabbit radial bone defect model was employed to evaluate various repair approaches, revealing that IM combined with the PLA-HA scaffold and eBM achieved bone regeneration efficiency comparable to IM with iliac crest bone grafts (ICBGs). These results indicate that PLA-HA with eBM and IM holds promise as a viable alternative to traditional autografts for repairing extensive bone defects. Kang et al. developed 3D-printed composite scaffolds made of poly (trimethylene carbonate) (PTMC), poly(L-lactic acid) (PLA), and hydroxyapatite (HA) (PTMC/PLA/HA and PTMC/HA) for bone regeneration applications [92]. These scaffolds exhibited high biodegradability and biocompatibility, effectively supporting osteoblast (MC3T3-E1) cell adhesion, proliferation, and differentiation in both in vitro and in vivo studies. The inclusion of hydroxyapatite (HA) enhanced osteogenic activity, while poly (L-lactic acid) (PLA) in PTMC/PLA/HA composites improved mechanical strength compared to PTMC/HA scaffolds. Specifically, PTMC/PLA/25%HA scaffolds exhibited lower cytotoxicity and superior cell proliferation relative to PTMC/25%HA variants, attributed to optimized material interactions and 3D-printed porous structures. After 8 weeks in a femoral defect model, PLA/PTMC/HA scaffolds showed a reduced bone volume fraction (BV/TV: ~13%) compared to PTMC/HA scaffolds (~16%), suggesting that PLA incorporation might slow degradation or interfere with later-stage osteogenesis. Despite this, both scaffold types created an osteogenic-friendly microenvironment, supporting bone tissue formation without requiring secondary surgical removal due to their biodegradability. These results position PTMC-based composites as promising candidates for clinical bone repair, particularly in scenarios demanding tailored mechanical and biological performance.

*b. Poly(caprolactone) (PCL)*

PCL, originally synthesized in the 1930s, gained commercial interest due to its biodegradability and compatibility with biomedical applications. Although it was initially overshadowed by rapidly degrading polymers like polylactides and polyglycolides, PCL has regained prominence, particularly in tissue engineering. Its favorable rheological and viscoelastic properties, ease of scaffold fabrication, and cost-effective production have contributed to its renewed interest. With its rigidity and slow degradation, PCL is primarily utilized in hard tissue engineering, providing structural support for over six months before gradually degrading within approximately three years. This extended resorption period allows for sustained support during healing. PCL’s prior approval for use in drug delivery devices streamlines its regulatory pathway, making it an attractive option. A custom-designed PCL-based airway splint was 3D printed and successfully implanted under an emergency-use exemption from the Food and Drug Administration [93]. Radhakrishnan et al. successfully created antimicrobial 3D-printed scaffolds for bone tissue engineering by integrating silver nanoparticles (AgNps) into a PCL matrix [94]. The resulting PCL/AgNps scaffolds showed improved mechanical properties, enzymatic stability, and strong antimicrobial effects against *Escherichia coli*, through the generation of reactive oxygen species (ROS) and silver ion diffusion. These scaffolds were compatible with human osteoblast cells (hFOB) and displayed 80% degradation within 20 days. The results indicate that these multifunctional scaffolds, particularly the 3Ag_2_ variant, hold significant potential for bone tissue engineering, providing both structural support and infection resistance. Benjamin Ho et al. explored the development of PCL and polycaprolactone-carbon nanotube (PCL-CNT) composite scaffolds for tissue engineering through 3D printing [95]. The study examines how different concentrations of CNTs affect the thermal, mechanical, and biological properties of the scaffolds. The findings reveal that increasing the CNT content enhances the mechanical properties, including elastic modulus, hardness, and peak load, as demonstrated by nanoindentation tests. Thermal and crystallinity characteristics were analyzed using differential scanning calorimetry (DSC) and Raman spectroscopy. Biodegradation experiments in enzymatic media showed that the degradation rate is influenced by both the CNT content and the scaffold structure. Cell viability and proliferation tests with H9c2 cells indicated healthy cell attachment. The 1% CNT concentration proved to offer optimal conductivity and stiffness for supporting cell growth, positioning these PCL-CNT nanocomposite scaffolds as strong candidates for cardiac tissue engineering, offering tunable biodegradation and good cell compatibility. Muwaffak et al. explore the rising problem of antibiotic-resistant wound infections by integrating antimicrobial metals such as zinc, copper, and silver into PCL for 3D-printed wound dressings [96]. These metals were selected for their broad antimicrobial effects, with copper and zinc also aiding the wound healing process. Using 3D scanning technology, they created customized dressings that fit the shape and size of individual patient wounds. The filaments containing these metals were made using hot melt extrusion and analyzed for their release characteristics. The dressings displayed quick antimicrobial release within 24 h, followed by a slower release extending up to 72 h. Antibacterial tests confirmed that the silver and copper-based dressings were most effective against *Staphylococcus aureus*, a common bacterium causing skin infections. This research demonstrates the potential of 3D printing and scanning technologies to create personalized, antimicrobial wound dressings tailored to specific patient requirements.

*c. Poly(propylene fumarate) PPF*

Poly(propylene fumarate) (PPF) is a biodegradable polymer extensively utilized in tissue engineering, especially for 3D-printed scaffolds aimed at bone and cartilage regeneration. Its distinct fumarate groups facilitate crosslinking, creating a durable network structure. PPF is well-suited for 3D printing methods such as SLA and FDM, enabling precise control over scaffold design. In SLA, diethyl fumarate (DEF) is incorporated to regulate viscosity and ensure smooth layering, though excessive amounts can compromise mechanical strength [97]. With adjustable mechanical properties, porosity, and flexibility, PPF scaffolds hold great potential for tissue repair and regeneration. In a recent study, Dadsetan et al. investigate the use of calcium phosphate coatings and recombinant human bone morphogenetic protein-2 (rhBMP-2) delivery to improve bone regeneration in biodegradable poly(propylene fumarate) (PPF) scaffolds [98]. Three types of calcium phosphate coatings—magnesium-substituted β-tricalcium phosphate (β-TCMP), carbonated hydroxyapatite (SBM), and biphasic calcium phosphate (BCP)—were applied to porous PPF scaffolds and evaluated in a rabbit calvarial defect model. The findings indicate that these coatings effectively sustained rhBMP-2 release while preserving scaffold porosity. After six weeks, micro-CT and mechanical testing showed that SBM-coated scaffolds facilitated the most significant bone formation, while β-TCMP and SBM coatings with rhBMP-2 delivery improved osteoconductivity and osteointegration even at lower rhBMP-2 doses. Conversely, BCP-coated scaffolds did not show notable benefits in bone regeneration. This study underscores the potential of calcium phosphate-coated PPF scaffolds as an effective strategy for repairing large bone defects. Luo et al. emphasize the significant influence of physico-chemical parameters on the cDLP 3D printing of poly (propylene fumarate) (PPF) resins for tissue engineering. By examining resin viscosity in relation to molecular mass, temperature, and solvent concentration, they developed an Arrhenius-based model to predict viscosity behavior. Their findings highlight the necessity of maintaining low viscosity to facilitate effective 3D printing, ensuring smooth resin flow and the proper mixing of additives. The 3D-printed PPF scaffolds demonstrated mechanical properties comparable to trabecular bone, reinforcing their potential for bone regeneration. Ongoing in vivo studies aim to assess the material’s degradation profile and tissue integration, advancing its biomedical applications.

*d. Polyether Ether Ketone (PEEK)*

**Figure 4:**
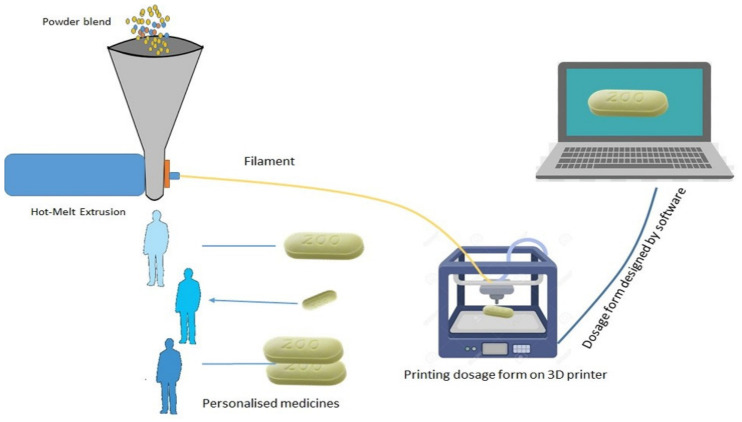
Diagram illustrating the applications of Hot-Melt Extrusion combined with FDM 3D printing for personalized drug delivery, reproduced permission from [102].

Polyetheretherketone (PEEK) has emerged as a highly valuable material in advanced manufacturing due to its exceptional mechanical strength, chemical and thermal resistance, and biocompatibility [99]. Its increasing use in aerospace, automotive, and biomedical applications highlights its versatility and reliability. In the medical field, PEEK custom cranial implants are gaining widespread adoption due to their superior biostability, radiolucency, and compatibility with standard fixation methods. The material’s modulus of elasticity closely matches that of cortical bone, minimizing stress shielding and making it a preferred alternative to metallic implants. PEEK implants can be precisely designed using CAD and fabricated through CNC machining or sintering techniques, enabling patient-specific solutions for cranial, frontal, malar, and mandibular defects [100]. Foletti et al. investigated the application of customized Polyetheretherketone (PEEK) implants for the aesthetic reconstruction of extensive cranial defects resulting from craniotomies. Conventional approaches, such as autologous grafts or surgical cement, often fall short in effectively restoring cranial symmetry. As a biocompatible and durable material, PEEK presents a promising alternative due to its lightweight nature, strength, and ability to protect the brain. The study highlights a case in which a patient underwent balloon scalp expansion before receiving a customized PEEK implant, achieving satisfactory aesthetic results with a straightforward postoperative recovery. Compared to titanium, PEEK is more manageable and offers an improved solution for addressing post-craniotomy deformities. Roskies et al. investigated a novel strategy to improve Polyetheretherketone (PEEK) for craniofacial reconstruction by altering its internal architecture into a trabecular network and incorporating mesenchymal stem cells (MSCs) [101]. Their study evaluated the interactions of bone-derived (BMSC) and adipose-derived (ADSC) stem cells with these enhanced structures. The findings revealed that while unmodified selective laser sintering (SLS)-printed PEEK facilitated the growth and integration of both cell types, ADSCs displayed more consistent osteogenic differentiation when cultured with the modified scaffolds. Clinical research has assessed the viability of 3D printing PEEK-based maxillofacial implants. This study underscores the potential of 3D-printed porous PEEK scaffolds in enhancing bone integration, offering a promising avenue for developing advanced load-bearing implants in craniofacial reconstruction. Tan et al. [102] developed PEEK filaments for drug delivery applications using hot melt extrusion technology, which could later serve as feedstock for FDM. See Figure 4 for the detailed setup. Their findings indicated that drug bioactivity and solubility were improved, enabling controlled and prolonged drug release. Several researchers have also designed and fabricated innovative scaffolds capable of delivering drugs in a precise and controlled manner. Future advancements in the 3D printing of soft materials, particularly lipid-based drug delivery systems, are expected to offer significant benefits in enhancing patient compliance. This underscores the need for further exploration of bioprinters and bioplotters.

#### 3.1.3. Composite Biomaterials

At first, only pure metals and polymers could be printed using 3D technology, but as the technology advanced, the creation of composite inks quickly gained popularity. These inks’ main goal is to enhance important characteristics such printability, processability, mechanical strength (stiffness), and bioactivity in order to support tissue integration and cellular function. Three primary categories can be used to classify the most popular composite ink systems for tissue engineering: ceramic, hydrogel, and polymer-based composites. Ceramics, biomolecules, carbon nanotubes, and, in rare instances, metals are some of the additives that are incorporated into these systems.

*a. Polymer—based composite*

Biodegradable polymers have emerged as a promising alternative to non-biodegradable materials in tissue engineering, regenerative medicine, and wound healing due to their biocompatibility, controlled degradation, and adaptability. While natural biodegradable polymers offer excellent biocompatibility, their mechanical limitations pose challenges in biomedical applications. Synthetic biodegradable polymers, such as PCL, PLA, poly(lactide-co-glycolide) (PLGA), and PEG, provide cost-effective solutions with tunable properties, making them ideal for scaffold fabrication [103]. Their chemical versatility allows for further design modifications, enhancing their clinical potential. The United States Food and Drug Administration (FDA) approved synthetic biopolymers to ensure safe degradation within the body, supporting their biomedical applications. Some durable synthetic polymers, like PMMA, face challenges such as aseptic loosening and bone tissue damage due to mechanical mismatches. Zhang et al. investigate the fabrication of (polylactic acid/nano-hydroxyapatite) PLA/nHA composite scaffolds for bone repair using cost-effective and reliable FDM 3D printing technology [104]. Their findings indicate that a high nHA content significantly improves the mechanical strength, bioactivity, and degradation characteristics of the scaffolds, making them superior to pure HA ceramic and cancellous bone. The study further reveals that a 50% nHA composition ensures optimal printability, structural precision, and osteogenic performance. The scaffolds facilitate in vitro bone-like apatite formation and promote in vivo osteo-regeneration. This research underscores the potential of FDM 3D printing as an affordable and effective method for producing personalized bone repair biomaterials with adjustable mechanical and biological properties for clinical use. In another study, Wang et al. explore the potential of 3D-printed PCL/bioactive glass (PCL/BG) composite scaffolds for bone repair [105]. While PCL possesses excellent biocompatibility and mechanical properties, it lacks inherent bioactivity. The incorporation of bioactive glass (5, 10, and 20 wt%) significantly improved the scaffolds’ hydrophilicity, enhanced cell adhesion and proliferation, and facilitated osteogenic differentiation by releasing Ca^2+^, P^5+^, and Si^4+^ ions. In vivo studies demonstrated that scaffolds with a higher bioactive glass content, particularly PCL@BG20, exhibited the greatest bone regeneration. FDM 3D-printed PCL/bioactive glass scaffolds offer a promising solution for personalized bone defect repair in clinical applications. Teixeira et al. highlights that the bioactivity of 3D-printed PLA scaffolds can be greatly improved through surface modification with polydopamine (PDA) and type I collagen (COL I) coatings [106]. Since synthetic polymers used in 3D printing generally lack bioactivity, this straightforward and cost-effective method enhances cellular interactions without the need for complex processing. The PDA layer increased COL I immobilization by 92%, resulting in improved early-stage cell adhesion, extracellular matrix deposition, and alkaline phosphatase expression, which signifies enhanced osteoinductivity. This single-step surface modification approach presents a valuable strategy for enhancing biomolecule adhesion in tissue engineering, especially for bone regeneration applications. Another study by Li et al. investigated the role of pore size in 3D-printed porous bioactive ceramic scaffolds for bone defect repair, as foreign body response (FBR) can influence tissue regeneration [107]. Their study on polycaprolactone/polyethylene glycol/hydroxyapatite (PCL/PEG/HA) scaffolds found that those with 600 μm pores (P600) significantly reduced a foreign body response (FBR), enhanced M2 macrophage polarization, facilitated vascular ingrowth, and promoted bone formation compared to smaller pores. The involvement of MyD88 protein in macrophage polarization was also suggested. While larger pores improve regeneration, they weaken mechanical strength, necessitating a balance. P600 scaffolds show strong potential for bone repair, but further research on pore size effects is required. The STL designs and corresponding 3D-printed PLCL and hydrogel-PLCL scaffolds, demonstrating high printing accuracy. SEM images reveal uniformly sized, highly interconnected porous structures, with comparable pore sizes and strut thicknesses in both scaffold types [108].

*b. Ceramic-based composite*

Ceramic composite biomaterials are extensively utilized in biomedical applications due to their exceptional stiffness, bioactivity, and resemblance to natural bone, making them suitable for orthopedic and dental procedures [109]. Directly 3D printing ceramics remains challenging due to their high melting points and limited light responsiveness. While powder bed (PB) and inkjet printing offer promising solutions, integrating ceramics into composite materials enables the use of more accessible techniques like FDM and SLA. Among ceramic-based biomaterials, biphasic calcium phosphate (a blend of HA and TCP) promotes cell viability and proliferation, though its mechanical properties remain weaker than natural bone [110]. To overcome this limitation, polymer additives such as PLGA and PVA have been introduced to enhance scaffold strength and flexibility. Continued advancements in material optimization, porosity regulation, and composite design will further improve ceramic-based scaffolds for bone regeneration, potentially serving as viable alternatives to autologous bone grafts. Mondal et al. highlight the importance of optimizing printing orientation to enhance the mechanical strength and bioactivity of 3D-printed scaffolds for biomedical applications [27]. This study examined the effects of different printing angles (0°, 45°, and 90°) on the mechanical properties of PLA and hydroxyapatite-modified PLA (PLA-HAp) scaffolds. Finite element modeling determined that scaffolds printed at a 90° orientation exhibited the highest compressive strength, making them well-suited for load-bearing bone tissue applications. Pure PLA scaffolds demonstrated poor cell attachment and proliferation, necessitating post-fabrication modification with nano-HAp. This enhancement significantly improved cellular interactions by promoting protein adsorption, which facilitated cell attachment and proliferation. Incorporating nano-HAp increased compressive strength by up to 47.16%, with a peak value of ~53 MPa and a porosity of ~47% for 90–oriented scaffolds. Sophie et al. provide a comprehensive analysis of 3D-printed bone tissue scaffolds composed of hydroxyapatite (HA) and poly(vinyl) alcohol (PVOH) composite powders, emphasizing the impact of precursor flowability on scaffold characteristics [111]. The study demonstrates that print orientation significantly influences mechanical strength, microstructure, and porosity, with *Y*-axis printed scaffolds exhibiting the highest compressive strength of 0.88 ± 0.02 MPa. A balance must be maintained between pore size, porosity, and sintering behavior, as *Y*-axis scaffolds retained some PVOH degradation residues. The research highlights the benefits of non-designed porosity and surface roughness in promoting osteoconduction and osteointegration. While the findings are promising, further refinement of precursor flowability and CAD design is necessary to improve the mechanical properties of scaffolds for bone tissue engineering.

*c. Hydrogel—based composite*

Hydrogels are hydrophilic polymeric materials, either physically or chemically crosslinked, that are widely recognized for their exceptional ability to absorb and retain large amounts of water [112]. By adjusting chemical composition, crosslinking density, and network structure, their properties can be tailored for specific uses. Hydrogels can also be modified to enhance cellular attachment or serve as stimulus-responsive drug delivery systems. While natural hydrogels offer biocompatibility but lack consistency, synthetic ones provide reproducibility but may have biocompatibility concerns due to harmful residues from manufacturing processes [112]. Hydrogels play a crucial role in bio-fabrication, injectable materials, and 3D cell culture applications. Lee et al. examined a composite made from alginate and collagen combined with silica particles [113]. The alginate–collagen composite exhibited superior mechanical properties compared to the individual components. Silica-infused alginate/collagen scaffolds were prepared by mixing solutions and then dip-coated with a silica solution. These coated scaffolds demonstrated better resistance to degradation, an increased compressive modulus, higher protein adsorption, and enhanced proliferation and ALP activity of seeded mouse pre-osteoblast cells (MC3T3-E1). These improvements are likely due to the Si-induced mineralization of the scaffold. The formation of an apatite layer on the surface helps prevent degradation. The observed increased ALP activity in the MC3T3-E1 cells may be attributed to the combined effects of the apatite layer and the release of silica ions, promoting osteoblastic activity. The hydrogels commonly used in biomedical applications are typically non-conductive to thermal, optical, and electrical stimuli since they are composed of insulating polymers. There is an increasing demand for conductive hydrogels for applications such as biosensors, actuators, and tissue engineering scaffolds. As a result, carbon-based nanomaterials (CBNs) are being explored as promising candidates for developing conductive, stimuli-responsive hydrogels. For instance, Zhang et al. created an actuator responsive to electrical and pH stimuli using a graphene–polyacrylamide (GO/PAM) composite hydrogel [114]. Similarly, Shin et al. enhanced the electrical conductivity of methacrylated gelatin (GelMA) hydrogel by incorporating carbon nanotubes (CNTs) [115]. The CNT-GelMA composite hydrogel was successfully employed as a tissue engineering scaffold, facilitating the improved electrophysiological function of cardiomyocytes (CMs). As a definitive assessment of angiogenic performance, GelMA and PRP-GelMA scaffolds were evaluated using the chick chorioallantoic membrane (CAM) assay, a widely recognized model for probing the angiogenic potential of biomaterials. In comparison with PRP-free GelMA, the PRP-GelMA scaffolds induced a markedly higher degree of vascularization in the surrounding region, with statistical significance (*p* < 0.0037) [116].

### 3.2. Properties of Biomaterials

Hydrogels, known for their remarkable water absorption and biocompatibility, have already proven effective in areas such as tissue engineering, drug delivery, and wound healing [117]. When combined with 3D printing technology, their potential is significantly expanded. This integration creates a powerful synergy that combines the precision of digital design with the adaptability and responsiveness of hydrogels, unlocking new possibilities. For example, Davood and colleagues introduce PETG-Fe_3_O_4_ nanocomposites for 4D printing, showing that lower concentrations of Fe_3_O_4_ nanoparticles enhance shape memory effects while preserving mechanical properties, offering a promising alternative to PLA-based smart materials for diverse applications [118]. Similarly, Mirasadi and c-workers present innovative PETG-ABS-Fe_3_O_4_ nanocomposites with great potential for 3D printing, highlighting the improvement in shape-memory performance due to iron oxide incorporation, which boosts heat transfer and elasticity, facilitating more efficient shape recovery under heat and magnetic fields [7]. Hydrogel-based 3D printing is making significant strides in many areas of medicine. Biocompatible hydrogels are being used by researchers to develop tissue scaffolds, artificial organs, and implants tailored to individual patients. Hydrogel structures’ shape, porosity, and mechanical properties can be carefully controlled, allowing for the highly accurate production of complicated anatomical shape scaffolds that mirror the composition and characteristics of natural tissues [119]. These bio-printed hydrogel scaffolds can support cell expansion, direct tissue regeneration, and aid in the formation of useful designed organs and tissues [120].

Due to the flexibility of PCL and the rigidity of PLA, the two materials are frequently combined to enhance their shape memory properties and mechanical performance. The distinct phase transition temperatures of PLA and PCL contribute to the shape memory behavior of their composite system. For instance, PLA nanofibers were incorporated between PCL polymer matrices to introduce intelligent functionality, resulting in the creation of a new variable nanoporous intelligent scaffold [121].

Biocompatibility is a critical requirement for biomaterials used in medical applications. Three-dimensional-printed MOF-based supramolecular hydrogels undergo comprehensive biocompatibility evaluations to ensure they do not trigger harmful biological reactions. These evaluations include both in vivo and in vitro testing, assessing factors like cell viability, proliferation, and inflammatory responses. By modifying the porosity and surface chemistry of the 3D-printed MOFs, these materials can improve interactions with biological tissues, reduce cytotoxicity, and enhance cell adhesion [122]. The porosity and surface characteristics of MOF-based supramolecular hydrogels can be tailored to promote biological interactions, supporting cell adhesion, proliferation, and differentiation. The hierarchical structure of MOFs within the hydrogel matrix mimics the ECM, aiding in nutrient exchange and facilitating tissue regeneration. Bioceramics are recognized for their biological activity, ability to promote bone tissue growth, and resorbable nature, making them key materials for bionic bone scaffolds [123]. β-TCP and HAp are the most commonly used bioceramics because they closely resemble the inorganic mineralization phase of bone [124]. Bone scaffolds require specific porosity and structure to replicate the properties of the bone they aim to replace. CaP ceramic scaffolds produced by traditional methods struggle to achieve intricate and precise structures, particularly in controlling pore diameter and size distribution with high accuracy [125]. To overcome this challenge, digital light processing (DLP) 3D printing has become widely used to create high-precision, customized bone scaffolds in recent years. Wu et al. introduced a closed-loop approach for producing high-precision, low-defect CaP ceramics by optimizing ceramic slurries, printing parameters, and post-processing techniques using the traditional method, contrasted with the regularly distributed and highly precise pore structure achieved by DLP [126].

## 4. Mechanical Predictions in Bioprinting

Scaffold design is an essential task for bioprinting. The design parameters are significantly affected both mechanical properties and cell behaviors [127]. Based on the design requirements, the model shape and size are formed by using the CAD software. These models are simulated by computer-aided engineering (CAE) software to find out the suitable parameters. The key design parameters are boundary shape, pore size, porosity and interconnectivity [128]. The suitable model is exported to the stereolithography (STL) file format, which is used for 3D printer. The published research on mechanical design can be categorized into two directions including (1) conventional design; (2) topology optimization; and (3) artificial intelligent (AI)-aided design.

### 4.1. Conventional Design

**Figure 5:**
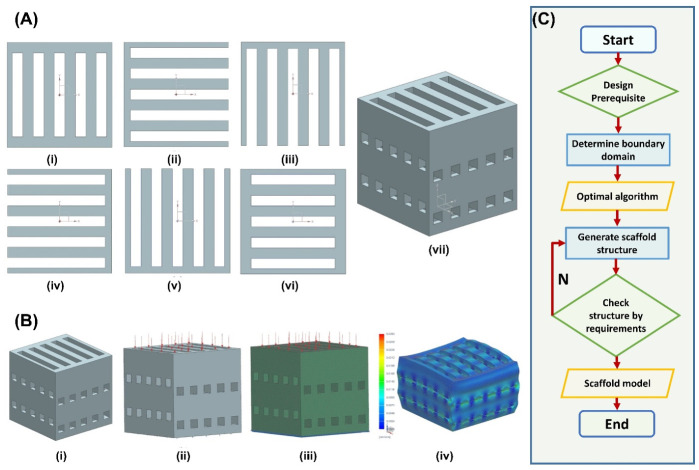
(**A**) The cuboid scaffold of 4 × 4 × 4 mm^3^. (**B**) The von Mises stress–strain distributions in the shear test simulated by Finite element method (FEM) in two lateral directions (x, y), reproduced with permission from [65]. (**C**) The scaffold design flowchart using the topology optimization.

To illustrate the conventional design process, the cuboid scaffold of 4 × 4 × 4 mm^3^ is created, as shown in Figure 5A. The thickness of each layer is 0.4 mm, which indicates this model contains 10 layers. Figure 5(Ai) shows the subtraction pattern of the first layer. Figure 5(Aii) shows the subtraction direction of the 2nd and 6th layers that is 0 degrees along the *x*-axis. The subtraction directions of the 3 remaining pair layers (3rd and 7th; 4th and 8th; and 5th and 9th) are 90 degrees, 180 degrees, and 270 degrees along the *x*-axis, as shown in Figure 5(Aiii), Figure 5(Aiv), and Figure 5(Av), respectively. Figure 5(Avi) presents the structure of the last layer. Finally, these layers are assembled to create the scaffold, as shown in Figure 5(Avii).

The conventional design method generates the scaffold library, which contains some models with differential architecture. Due to the difference in inner geometry and pattern orientation, the mechanical behaviors of each model that influence cell immigration and ingrowth are different [129]. Therefore, the mechanical behaviors of the scaffold are the basic parameters of the targeted tissue [130]. To create a suitable model, the finite element method (FEM) method is used to simulate the mechanical properties of the models created by using CAD software. The FEM is a numerical method that can predict the mechanical behaviors applied to design 3D (three-dimensional) scaffold architecture [131]. Mechanical behaviors are considered as strength, stiffness or elasticity. FEM analysis demonstrated that scaffold geometry strongly influences compressive mechanical behavior, with vertical supportive columns playing a key role in load resistance. Scaffolds containing thicker or more continuous columns exhibited enhanced compressive stiffness, while the absence of such supportive features resulted in reduced stress concentration and mechanical resistance. The simulations confirm that internal architectural design is a critical factor in governing the mechanical performance of printed scaffolds under compression.

The compressive elastic modulus is determined to evaluate the mechanical behaviors of the scaffolds [132]. Using CAE software, the FEM is conducted to measure the compressive modulus of the scaffold [133]. All scaffold models from the CAD library are simulated to find out a suitable model. Figure 5B shows the simulation processes. First, the 3D CAD of the scaffold model is imported to the CAE environment to prepare the FEM model. Second, the material and boundary conditions are applied to the FEM model. In this step, the bottom surface is fixed, and the compressive force is applied to the top surface. Third, the nodes and elements are generated by meshing. The mesh size significantly affects the simulation time. The simulation time is increased when the mesh size decreases and vice versa. Due to the dependency of the FEM result accuracy on the mesh size, the number of nodes and elements should be optimized to save simulation time. Fourth, the results are plotted as stress–strain information after running the simulation stage. Finally, the suitable scaffold is obtained by comparing the simulation result of each model.

### 4.2. Topology Optimization

To replicate the biological characteristics of native scaffold–tissue systems, scaffold design must ensure a smooth biomechanical transition between the scaffold and surrounding tissue, which inherently increases design complexity. Such designs must simultaneously satisfy mechanical stiffness and efficient fluid transport requirements [134]. Topology optimization offers a promising strategy to address these competing demands. This approach systematically optimizes material distribution within a prescribed design domain using mathematical algorithms [135]. In topology optimization, material density varies continuously from 0 to 1, representing void regions and fully solid material, respectively. According to homogenization theory, the evolution of material density within the domain Ω governs the resulting structural configuration. A density of 1 indicates a fully solid domain, whereas reducing the density to intermediate values (e.g., 0.5) leads to the formation of void regions, depicted as black areas. The final porous architecture is thus generated through the optimization algorithm in accordance with predefined design constraints and performance requirements.

The topology optimization technology is exploited to design the scaffold to meet the requirements of elasticity modulus and permeability [136]. Figure 5C shows the scaffold design flowchart by using topology optimization. First, the boundary domain is defined as a full solid. Second, the optimal algorithm is applied to generate the porous architecture. Third, the scaffold structure is checked by the design requirements. Finally, the scaffold model is obtained in STL format. The optimization algorithm is selected based on the design prerequisites. The exploitation and inverse homogenization algorithms offer a potential alternative to design scaffolds. The mechanobiological algorithm is used for remodeling design. The stiffness optimization is exploited to provide a biomimetic environment for restoration [137]. Based on the bulk modulus and diffusivity properties, the multi-objective algorithm is adapted to optimize microstructure design [138]. In addition, various works present the algorithm to optimize the fluid transport properties. The permeability properties of scaffold are analyzed using a 2nd order different equation obtained by the Darcy Law and the balance steady state flow [139]. The homogenization of Darcy’s Law is an approach to optimize the scaffold design following the permeability criteria [140]. The algorithm based on Stock flow is used to minimize the power dissipation to optimize the material distribution of the scaffold [141].

### 4.3. Artificial Intelligent (AI) Aided Design

To mimic the biomimetic behaviors of original tissue, scaffold design remains a challenging task due to the geometrical complexity and large aspect ratio. It is difficult to design the complex geometry by using conventional design approaches and topology optimization. The simulation time is also significantly increased when the FEM is applied to simulate the complex geometry. To solve these problems, AI and ML are innovative approaches for deciphering the complexity of scaffold design. The key support for AI and ML in the scaffold design is predictive modeling. Using AI and ML algorithms, computers learn from extensive data and make predictions. Based on the dataset training, the scaffold is developed for enhanced biomimetic behaviors. AI and ML have been applied to predict the vascularization of the scaffold in repair strategies [142], and associate in vitro performance with physico-chemical characteristics [143].

There are several AI and ML strategies that are used to predict the scaffold model. Computer-supported learning has been developed to design scaffold frameworks. Using image datasets as input, 2D convolutional neural networks (CNNs) are used to predict multiple characteristics of porous materials, which is an approach for designing scaffolds. Using 3D CNNs is a novel method for predicting the mechanical properties of complex scaffold structures. In this case, diagnostic behaviors are obtained using medical images as input [144].

## 5. Biomedical Application

### 5.1. Tissue Engineering

Three-dimensional printing, an advanced additive manufacturing technique, facilitates the fabrication of modular, patient-specific scaffolds with complex structural designs and high adaptability. This method enables the construction of models derived from tissue images obtained through commonly used medical imaging technologies such as computed tomography (CT) and magnetic resonance imaging (MRI), a capability that conventional fabrication techniques lack. In recent years, 3D printing has gained widespread application in the medical field, including its use in craniofacial implants, dental molds, crowns, and implants, prosthetic components, on-demand medical tools, surgical models, scaffolds for tissue regeneration in skin and bone, organ printing, and tissue models for drug discovery. The ability to design patient-specific devices, regulate scaffold porosity and orientation, and integrate various materials—both synthetic and biological—has garnered significant attention. Consequently, this technological advancement has led to pioneering medical treatments and devices. Recent studies have highlighted the potential of 3D printing in bio-fabricating anatomically precise tissue constructs with structural integrity. These advancements are largely driven by ongoing progress in 3D printing technology and biomaterial innovation.

*a. Regenerative medicine*

Regenerative medicine involves the creation of medical tools and devices designed to restore and repair damaged or malfunctioning tissues and organs. An optimal scaffold is composed of biocompatible and biodegradable materials, featuring a biomimetic 3D porous structure and biomechanical properties that align with the host tissue. Chou et al. explored 3D-printed Fe–Mn biodegradable scaffolds for bone repair. The scaffolds, with 36.3% porosity, exhibited similar mechanical properties to natural bone and corroded faster than pure iron, reducing stress shielding. In vitro tests showed good cell compatibility and infiltration. The findings suggest that 3D-printed Fe–Mn alloys are promising for craniofacial applications, with potential for improved biodegradability. Liu et al. developed a method for creating titanium bone scaffolds using rapid prototyping (RP) with selective laser sintering (SLS) [145]. By optimizing laser parameters, scaffolds were produced in 3 h, with post-treatment boosting compressive strength to 142 MPa. This process shows great potential for fabricating complex bio-metal scaffolds for tissue engineering. Bioceramic materials, including ceramic composites, amorphous glasses, and crystalline ceramics, are regarded as promising candidates for bone tissue engineering (B-TE) applications due to their excellent corrosion resistance, mechanical properties, and high compressive strength. A widely studied bioceramic, beta-tri-calcium phosphate (β-TCP), has been combined with hydroxyapatite (HA) to create porous scaffolds for bone repair, as investigated by Ishack et al. [146]. Collagen-based open-cell porous scaffolds with interconnected channels have been fabricated using indirect 3D printing and freeze-drying methods. Combining indirect 3D printing with foaming techniques has resulted in highly porous gelatin scaffolds with intricate channel architectures. The use of 3D printing in various medical surgeries, including dental, neurosurgery, maxillofacial, orthopedic, plastic, and reconstructive surgeries, has been successfully demonstrated by Malik et al. [147] and Klein et al. [148]. Ayoub et al. [149] presented a technique for producing precise composite 3D-printed mandible models with plaster teeth, which serve as a dependable tool for preoperative diagnosis and surgical planning. These models exhibit minimal errors, staying within clinically acceptable limits, and improve the planning of complex craniofacial surgeries by offering accurate depictions of both bone structures and teeth. This method provides advantages over conventional approaches, including tactile feedback for occlusal interdigitation, enhancing surgical accuracy, shortening operation times, and helping patients better understand the procedure. Louvrier et al. suggested that in dental implant surgeries, the most commonly printed 3D devices are surgical guides, which are specifically designed to assist in the precise direction and placement of implants during drilling and fitting. Skardal et al. explored the potential for skin regeneration in mouse skin wounds using printed hydrogels incorporated with amniotic fluid-derived stem (AFS) cells [150]. They created hydrogel composites by mixing fibrinogen and collagen in a 50:50 volume ratio, along with AFS cells and mesenchymal stem cells (MSCs). Using an inkjet 3D printer, the fibrinogen/collagen hydrogel composites with cells and thrombin were printed directly onto the skin wounds of nude mice, layer by layer. Wounds treated with these composites containing AFS and MSC cells showed improved wound closure and re-epithelialization over 14 days, with increased vessel density and larger capillary diameters compared to the fibrin/collagen gel alone.

*b. Organs-on-a-chip*

An emerging area in tissue engineering focuses on developing functional tissues for use in drug screening and disease modeling. Drug activity is evaluated in 2D cultures, animal models, and clinical trials as part of current drug screening techniques [151]. Nonetheless, it is becoming more well recognized that 2D cultures are unable to reproduce the interactions between cells and between cells and the matrix that are essential for regular cellular activity [152]. For in vitro studies to be successful, the platform must mimic the in vivo environment by using 3D cell cultures in biomaterials with ECM, incorporating a perfusable network to simulate circulation, and providing mechanical and electrical stimulation when necessary to maintain normal cellular activity. Over the past decade, the concept of organs-on-a-chip, as miniaturized in vitro systems that integrate human cells to reproduce the structural, biochemical, mechanical, and functional characteristics of real organs, has emerged. These systems offer a promising bridge between in vitro experiments and clinical trials, delivering more physiologically relevant results for high-throughput drug screening compared to traditional animal models. Researchers have already engineered miniaturized versions of several organs, including the liver, lung, intestine, kidney, and heart tissue [153]. Huh et al. developed a biomimetic lung-on-a-chip model that replicates the alveolar–capillary interface and key organ-level responses to pathogens and inflammatory stimuli [154]. The upper compartment contained alveolar cells, while the lower compartment housed pulmonary cells, effectively simulating the alveolar–capillary barrier. To mimic respiratory movements, the system operated under a vacuum, allowing alveolar expansion and contraction. Inflammatory responses were introduced by delivering neutrophils through fluidic channels. Their findings demonstrated that mechanical strain intensifies the lung’s reaction to nanoparticles, promoting their uptake and transport across tissue layers. This study highlights the potential of dynamic organ-on-a-chip systems as advanced in vitro models for drug screening and toxicology, offering a cost-effective alternative to animal and clinical studies. Jang et al. developed a kidney-on-a-chip model that serves as an advanced in vitro platform for studying renal physiology, disease mechanisms, and drug-induced nephrotoxicity [155]. By incorporating human kidney proximal tubular epithelial cells into a microfluidic system, this model simulates essential renal functions such as glucose reabsorption, albumin transport, and enzyme activity under physiological shear stress. Unlike conventional static cultures, the dynamic environment promotes cell polarization and primary cilia formation, enabling more precise nephrotoxicity predictions. This technology offers a cost-effective and human-relevant alternative for preclinical drug testing, enhancing the accuracy of kidney disease research. A key challenge in drug discovery is the low predictive accuracy of in vitro toxicity assays, largely due to their inability to replicate pharmacokinetics (PK). Although mathematical PK-PD models are available, their limitations restrict widespread use. To address this, Sung et al. developed a novel microscale cell culture analog (µCCA), a microfluidic system that connects multiple cell culture chambers to simulate multi-organ interactions and assess drug toxicity in a pharmacokinetic-based manner [152]. The design incorporates gravity-induced flow for pumpless operation and supports hydrogel-based cell cultures. Experiments with liver, tumor, and marrow cells exposed to 5-fluorouracil revealed distinct cellular responses compared to static cultures. By integrating PK–PD modeling with in vitro microfluidic testing, this approach enhances drug toxicity prediction and provides deeper insights into drug mechanisms. Agarwal et al. created heart muscle-on-a-chip models using alginate hydrogels with microgrooves. These micropatterned substrates promoted higher cellular activity compared to flat hydrogels. Alginate is suitable for muscle tissue engineering but lacks protein adhesion and patterning capabilities. To address this, they used microcontact printing for chemical guidance and micromolding for topographical cues, enabling anisotropic muscle tissue formation. These techniques allow for functional contractility assays, making micropatterned alginate hydrogels a versatile platform for in vitro muscle models and tissue engineering. Recent progress in microfluidics, micro electromechanical systems (MEMS), and biomaterials has made the development of a body-on-a-chip, designed to replace animal models in drug testing, more feasible.

### 5.2. Drug Delivery Systems

Three-dimensional-printed scaffolds for localized drug release represent a promising approach in drug delivery systems. These scaffolds can be precisely engineered to control the spatial and temporal release of therapeutic agents, offering targeted treatment directly at the site of interest. By utilizing advanced 3D printing techniques, the scaffolds can be tailored to match the desired drug release profiles and conform to specific anatomical structures. This localized approach improves drug efficacy, minimizes systemic side effects, and enhances patient outcomes, making it a valuable strategy in personalized medicine and regenerative therapies. Table 3 provides an overview of key studies that explore the use of 3D-printed scaffolds integrated with nanoparticles for bone cancer treatment. Recently, 3D-printed programmable release capsules made of a GelMA matrix, containing growth factors and encased in a PLGA shell functionalized with gold nanorods, were developed for stimuli-triggered core/shell release systems. Meng et al. developed 3D in vitro tumor models that replicate native tumor microenvironments and can enhance the effectiveness of anticancer drug testing [156]. Using 3D bioprinting, tumor constructs are created by precisely positioning living cells, biomaterials, and programmable release capsules, enabling controlled molecular signaling. Vascularized tumor models simulate key cancer dissemination processes, such as invasion, intravasation, and angiogenesis. These models provide a valuable platform for studying tumor progression and metastasis while serving as a tool for preclinical drug screening and therapeutic evaluation. Liu et al. presented a 3D-printed core/shell fiber scaffold designed for on-demand drug delivery and tissue regeneration [157]. By coating drug-loaded alginate–gelatin hydrogels with PCL and polydopamine (PDA), the system achieves controlled, sustained drug release while enabling near-infrared (NIR) laser-triggered release through photothermal effects. This dual-function approach effectively inhibits tumor growth and supports wound healing. The Gel/PCL/PDA scaffolds offer a promising platform for localized cancer therapy, particularly for post-surgical applications, by eliminating residual cancer cells and aiding tissue repair. A schematic illustration of the fabrication process of PDA-coated Gel/PCL core/shell scaffolds designed for NIR-triggered on-demand drug release, enabling tumor therapy and wound healing, was created. Wei et al. introduced an innovative strategy for localized cancer treatment using 3D-printed core-shell hydrogel fibers and scaffolds with NIR-responsive, on-demand drug release [158]. The design incorporates a polydopamine (PDA)/alginate shell and a drug-loaded, temperature-sensitive hydrogel core, enabling controlled drug delivery. Upon NIR irradiation, the photothermal properties of PDA trigger a gel–sol transition in the core, promoting targeted drug release. This approach effectively eradicates residual and recurrent breast cancer cells after surgery while allowing real-time monitoring via PA imaging. The engineered scaffolds offer significant potential for post-surgical breast cancer therapy and localized drug delivery applications.

### 5.3. Personalized Medicine

*a. Dose personalization*

Since pharmaceutical dosages vary based on age and body weight, a 3D printing platform enables precise dose customization, which is particularly beneficial for pediatric patients. Certain medications have a narrow therapeutic window, making it difficult to adjust dosages while ensuring patient safety. When produced in fixed strengths, these formulations pose significant challenges. In the case of enteric-coated tablets, issues such as dosage dumping, along with high costs and time-consuming manufacturing processes, may arise [175]. Pietrzak et al. introduced a customizable tablet dosing system utilizing FDM 3D printing integrated with Hot Melt Extrusion (HME) for precise and personalized drug administration [176]. This approach enables accurate dose adjustments (91–95% precision) using methacrylic and cellulose-based polymers while preserving drug stability. Although higher resolution increased printing time, it had little effect on drug release. The cost-effectiveness, compact design, and versatility of FDM 3D printers highlight their potential for future applications in personalized medicine and clinical settings. Okwuosa et al. investigated dual FDM 3D printing for creating customized gastric-resistant tablets, addressing challenges in personalized drug delivery [175]. Using polyvinylpyrrolidone (PVP) and methacrylic acid co-polymer for core-shell designs, they developed tablets with different shell thicknesses. A minimum thickness of 0.52 mm was required to ensure protection in acidic conditions, allowing for controlled drug release. The technique successfully integrated various drug candidates while preserving their stability. Despite some resolution limitations, FDM 3D printing proved to be a promising approach for manufacturing delayed-release tablets tailored to individual patients. Sjoholm et al. investigated semisolid extrusion 3D printing as a method for fabricating personalized warfarin orodispersible films (ODFs) to overcome dosing challenges and swallowing difficulties in pediatric and geriatric patients [177]. This technique successfully produced thin, transparent, and flexible films with precise drug content (3.9–7.4 mg) and strong linearity (R^2^ = 0.9996). The approach reduces contamination risks and shows potential for on-demand hospital compounding. Although the films exhibited appropriate pH and drug uniformity, further studies are required to refine drug release, stability, and storage for clinical implementation. With advancements in genetic sequencing, 3D printing could potentially be used to manufacture medications tailored to a patient’s genomic profile, biochemical processes, or physiological markers.

*b. Programmable release system*

Various rate-controlled polymers and additional fabrication features associated with the technique can be utilized to create programmable drug delivery systems. This customizable system, offering tailored release patterns for individual patients, is made possible through 3D printing technologies [178]. Martinez et al. showed that 3D printing (additive manufacturing) enables the production of tablets in different shapes, providing the opportunity to examine how geometry influences drug release [178]. Paracetamol-loaded printlets were created using stereolithographic 3D printing in shapes such as cubes, discs, pyramids, spheres, and tori, with variations in surface area (SA) and surface area/volume ratio (SA/V). Dissolution tests indicated that printlets with a constant SA/V ratio released the drug at a steady rate, while those with constant SA exhibited varying release rates. As the SA/V ratio increased, the dissolution rate also rose. These findings demonstrate that 3D printing can create personalized dosage forms with adjustable release rates, making it a promising approach for personalized medicine. Fu et al. explored the application of 3D printing to produce personalized progesterone-loaded vaginal rings with controlled release [179]. By utilizing FDM printing, the rings were crafted from a mixture of PLA, PCL, and PEG 4000, demonstrating sustained drug release for more than 7 days. This technology allows for customization, presenting a promising option for gynecological therapies, with potential for further improvements. Fang et al. utilized 3D printing technology to develop personalized, controlled-release glipizide formulations aimed at addressing the rising global prevalence of type II diabetes [180]. Their study focused on optimizing prescription adaptability, drug release control, and the customization of glipizide preparations. Using the semisolid extrusion (SSE) method in combination with traditional excipients, the approach enabled flexible dosage adjustments and streamlined automation. The results demonstrated that the formulations met the necessary performance standards, highlighting the potential of 3D printing for personalized drug delivery. The research explored the adaptability of this technology with other model drugs, reinforcing its broad application prospects in community healthcare and telemedicine.

## 6. Future Directions

The integration of artificial intelligence with 3D printing for automated design optimization and real-time process control. The development of multi-material printing systems for simultaneous deposition of cells, biomaterials, and bioactive molecules. The advancement of in situ bioprinting techniques for direct application in surgical environments. The improvement of vascularization strategies within bioprinted tissues using microfluidics and endothelial cell patterning. The expansion of printable bioinks with tunable mechanical, rheological, and degradation properties. The standardization of mechanical and biological characterization protocols for 3D-printed constructs. The enhancement of high-throughput organ-on-chip platforms for drug screening and toxicity testing. The design of closed-loop manufacturing systems for personalized pharmaceutical dosage forms based on patient-specific data.

Investigation of immunological responses and the long-term biocompatibility of implanted 3D-printed devices. The implementation of regulatory frameworks and quality control measures for clinical-grade bioprinted products. The miniaturization of body-on-a-chip models for comprehensive pharmacokinetic and pharmacodynamic assessments. The commercial scaling of cost-effective and GMP-compliant 3D printing platforms for clinical translation. The development of biodegradable electronic interfaces for integration with 3D-printed tissue scaffolds. The application of 4D printing to enable time-responsive and environmentally adaptive therapeutic systems.

## 7. Conclusions

This review highlights the critical advancements in biomaterials, mechanical design strategies, and predictive modeling that are driving 3D printing toward translational and clinical applications. Beyond structural support, bioprinted scaffolds have demonstrated significant potential in drug delivery studies, where scaffold drug retention and controlled release properties are essential for achieving therapeutic efficacy and minimizing systemic toxicity. The integration of intelligent biomaterials and programmable scaffold architectures now enables the design of stimuli-responsive platforms that provide spatiotemporal control over drug release, thereby improving localized treatment outcomes and advancing personalized medicine. In pharmaceutical applications, 3D printing further allows dose personalization, the development of controlled-release drug formulations, and the fabrication of implantable devices with integrated therapeutic functions. Several critical challenges persist, including the establishment of standardized mechanical and drug release evaluation protocols, scalable production strategies, long-term biocompatibility assessment, and regulatory harmonization. The integration of scaffold mechanics with drug retention and controlled release properties positions 3D printing as a powerful platform bridging biomedical engineering and pharmaceutical sciences. The continued advancement of this field holds significant promise for the progression of regenerative medicine, the enhancement of drug delivery systems, and the realization of personalized healthcare solutions.

## Figures and Tables

**Table 2:** Comparison of representative bioprintable biomaterials’ drug loading and release properties.

Material	Drug Loading	Release Profile	Mechanism	Crosslinking Effect	Ref.
Collagen (Type I)	Low to moderate, increasing with collagen concentration and network density	Initial burst release followed by short-term sustained release	Primarily diffusion-controlled, with a minor contribution from matrix remodeling or degradation at later stages	Riboflavin photocrosslinking raises G′ and network density, limiting burst release and slowing diffusion. The effect is weaker at high collagen levels. Too much crosslinking can lower drug loading and cell viability, while slightly alkaline pH (~8) stiffens the gel and helps control release.	[79]
GelMA (Type A&B)	Moderate to high, tunable by degree of substitution and polymer concentration; higher DS adds more functional groups and improves drug incorporation	Reduced burst release compared to gelatin; supports sustained release dependent on crosslinking density and offers higher stability than physically crosslinked hydrogels	Mostly diffusion-controlled, but at greater crosslinking concentrations and during prolonged culture, there may be a shift toward a diffusion–degradation hybrid mechanism	Photocrosslinking at 365 nm increases storage modulus and network density, effectively reducing burst release and slowing diffusion. Higher degrees of substitution (DS) and polymer concentrations lead to greater crosslink density, which promotes more sustained release. Type B GelMA typically exhibits stronger crosslinking owing to its higher DS. While tunable stiffness (0.1–150 kPa) allows for tailored release profiles, excessive crosslinking can impair diffusion and compromise cell viability	[80]
Alginate/Gelatin composite	Moderate–high; enhanced by gelatin (provides binding sites) while alginate contributes to physical entrapment; tunable via composition ratio	The combination provides a reduced burst release compared to pure gelatin, supports sustained release over extended periods, and offers greater stability than single-component hydrogels	Primarily diffusion-controlled through alginate network; gelatin introduces partial degradation-controlled contribution (enzymatic/thermal) → hybrid mechanism	Ionic crosslinking of alginate (e.g., Ca^2+^) increases network density → reduces burst release and slows diffusion; gelatin provides bioactivity but is less stable (can dissolve at physiological temperature), leading to increased porosity over time; overall release can be tuned via alginate:gelatin ratio and crosslinking density; higher crosslinking → slower release but may reduce diffusivity	[81]
AW/PLA	Low–moderate; primarily enabled through PLA phase (drug incorporation into polymer matrix); ceramic AW phase contributes minimally to loading but enhances bioactivity	Predominantly long-term sustained release with minimal burst; suitable for extended delivery due to slow degradation of PLA	Mainly degradation-controlled (PLA hydrolysis); secondary diffusion contribution through porous structure and interconnected networks	AW increases porosity, bioactivity, and tissue ingrowth, indirectly facilitating diffusion; strong interfacial bonding prevents delamination → ensures consistent release; PLA degradation rate controls release kinetics; no chemical crosslinking; instead controlled by thermal bonding and physical interlocking between AW and PLA → stable structure with slow resorption; tunable via porosity, phase distribution, and degradation rate	[82]

**Table 3:** An overview of material compositions, bioprinting methods, bioink crosslinking strategies, and the biological outcomes documented in 3D bioprinting research.

Crosslinking/Reticulation Strategy	Bioink Composition	Bioprinting Technique	Main Outcomes and Biological Performance	Ref.
Fibrinogen–thrombin reaction	PL	Not specified	The bioink effectively supported corneal tissue re-epithelialization under ex vivo conditions.	[159]
Thrombin-mediated and Ca^2+^-induced fibrinogen gelation	PL + cellulose nanocrystals	Single extrusion using dual reservoirs with a support bath	Enabled ASC bioprinting without animal-derived supplements, maintaining cell viability and print fidelity.	[160]
Ca^2+^-triggered alginate crosslinking	PRP + alginate	Single extrusion	Promoted MSC proliferation and migration, as well as vascular-like tube formation with HUVECs.	[161]
UV light-induced photocrosslinking	PLMA/PCL, PLMA/alginate, PLMA/gelatin	Dual extrusion	Multiple cell types were successfully printed, and the constructs retained their geometry following photocrosslinking.	[162]
No chemical crosslinking	Fragmented fibroblast cell sheets + agarose hydrogel	Dual extrusion	First report of a bioink based on fragmented cell sheets; printed filaments fused into 3D constructs, although structural evidence was limited.	[163]
No chemical crosslinking	Keratin + PCL	Single extrusion combined with electrospinning	A stratified epidermal–dermal architecture was achieved, leading to improved in vivo wound healing compared with PCL scaffolds alone.	[164]
No chemical crosslinking	DBM + PLG + HA	Single extrusion	HA/DBM composites showed comparable spinal fusion efficacy while eliciting reduced inflammatory cytokine responses relative to commercial grafts.	[165]
Temperature-responsive gelation	adECM integrated with a PCL framework	Dual extrusion	ASC-laden printed constructs significantly enhanced soft tissue regeneration after in vivo implantation.	[166]
UV photocrosslinking	PRP with varying GelMA ratios	Single extrusion using three distinct inks for multilayer printing	Encapsulated ASCs underwent composition-dependent differentiation toward osteochondral tissue.	[167]
Blue-light photocrosslinking	PRP + GelMA	Single extrusion	Enhanced angiogenesis was observed in vitro and in CAM assays without inducing myofibroblast differentiation.	[116]
Temperature-triggered gelation	adECM + type I collagen within a PCL framework	Dual extrusion	Promoted adipose tissue regeneration through increased neovascularization and tissue formation.	[108]
Ca^2+^ diffusion–based crosslinking	fECM or aECM + alginic acid	Single extrusion onto gelatin with CaCl_2_ support	Introduced supercritical fluid–extracted ECM bioinks; however, no fully developed 3D printed constructs were demonstrated.	[168]
Thrombin–fibrinogen crosslinking	adECM + animal-derived ECM	Single extrusion using removable PCL support	Generated constructs exhibiting hyaline cartilage–like osteochondral repair following removal of the PCL support.	[169]
Temperature-dependent gelation	Type I collagen	Single extrusion within a gelatin support bath	Xenogeneic collagen failed to produce mechanically stable 3D constructs compared with human-derived collagen; no 3D structures were obtained.	[170]
Thrombin embedded within the ink	Type I collagen + HA + blood plasma	Laser-assisted bioprinting (LAB)	First demonstration of LAB using human-derived materials, achieving high-resolution printing and robust ASC proliferation and migration in vitro and in vivo.	[171]
UV light photocrosslinking	PL + GelMA	Single extrusion	Supported dermal fibroblast adhesion, proliferation, and extracellular matrix deposition.	[172]
Temperature-responsive gelation	adECM combined with a PCL framework	Dual extrusion	First demonstration of the printability of human-derived decellularized matrices using a synthetic PCL support framework.	[32]
No chemical crosslinking	DBM + PLG + HA	Single extrusion	Composite scaffolds outperformed DBM alone in vivo, showing enhanced spinal fusion outcomes.	[173]
UV light photocrosslinking	Keratin + photosensitive resin	Digital light processing (DLP)	Printed hydrogels significantly improved wound healing outcomes in a porcine thermal burn model in vivo.	[174]

## Data Availability

No new data were created or analyzed in this study. Data sharing is not applicable to this article.

## Abbreviations

2PP	Two-Photon Polymerization
ABS	Acrylonitrile Butadiene Styrene
ADSC	Adipose-Derived Stem Cell
AFS	Amniotic Fluid-derived Stem cell
AgNps	Silver Nanoparticles
AI	Artificial Intelligence
ALP	Alkaline Phosphatase
AM	Additive Manufacturing
BCP	Biphasic Calcium Phosphate
BJT	Binder Jetting
BMSC	Bone Marrow-derived Mesenchymal Stem Cell
CAD	Computer-Aided Design
CAE	Computer-Aided Engineering
CAM	Computer-Aided Manufacturing (or Chorioallantoic Membrane)
CBNs	Carbon-Based Nanomaterials
CFD	Computational Fluid Dynamics
CLIP	Continuous Liquid Interface Production
CMs	Cardiomyocytes
CNC	Computer Numerical Control
CNNs	Convolutional Neural Networks
CNT	Carbon Nanotube
CT	Computed Tomography
dECM	Decellularized Extracellular Matrix
DEF	Diethyl Fumarate
DIW	Direct Ink Writing
DLP	Digital Light Processing
DSC	Differential Scanning Calorimetry
eBM	Enhanced Bone Marrow
ECM	Extracellular Matrix
FBR	Foreign Body Response
FDA	Food and Drug Administration
FDM	Fused Deposition Modeling
FFF	Fused Filament Fabrication
GAGs	Glycosaminoglycans
G-code	Geometric Code
GelMA	Gelatin Methacryloyl
GO/PAM	Graphene Oxide/Polyacrylamide
HA	Hydroxyapatite
hFOB	Human Fetal Osteoblast cells
HME	Hot Melt Extrusion
ICBG	Iliac Crest Bone Graft
IM	Induced Membrane
iPSCs	Induced Pluripotent Stem Cells
LAB	Laser-Assisted Bioprinting
MA-HF	Microalgae-Loaded Hollow Fibrous
MEX	Material Extrusion
ML	Machine Learning
MOF	Metal-Organic Framework
MRI	Magnetic Resonance Imaging
MSCs	Mesenchymal Stem Cells
nHA	Nano-Hydroxyapatite
PCL	Polycaprolactone
ODFs	Orodispersible Films
PA	Photoacoustic
PCL-CNT	Polycaprolactone-Carbon Nanotube composite
PDA	Polydopamine
PEDOT-PSS	Poly(3,4-ethylenedioxythiophene) Polystyrene Sulfonate
PEEK	Polyether Ether Ketone
PEG	Polyethylene Glycol
PETG	Polyethylene Terephthalate Glycol
PETG-Fe_3_O_4_	PETG with iron oxide nanoparticles
PK-PD	Pharmacokinetic-Pharmacodynamic
PLA	Polylactic Acid
PLA-HA	PLA-Hydroxyapatite composite
PLCL	Poly(L-lactide-co-caprolactone)
PLGA	Poly(lactide-co-glycolide)
PMMA	Poly(methyl methacrylate)
PPF	Poly(propylene fumarate)
PRP	Platelet-Rich Plasma
PTMC	Poly(trimethylene carbonate)
PVA	Polyvinyl Alcohol
PVOH	Poly(vinyl alcohol)
PVP	Polyvinylpyrrolidone
RGD	Arginine-Glycine-Aspartic acid
rhBMP-2	Recombinant Human Bone Morphogenetic Protein-2
SBM	Carbonated Hydroxyapatite
SLS	Selective Laser Sintering
SSE	Semisolid Extrusion
STL	Standard Triangle Language/Stereolithography file format
TPE	Thermoplastic Elastomer
TPU	Thermoplastic Polyurethane
β-TCMP	Magnesium-substituted β-tricalcium phosphate
β-TCP	Beta-tricalcium phosphate
µCCA	Microscale Cell Culture Analog
